# Editorial: Epigenetics and genomic causality in development and chromatin research

**DOI:** 10.3389/freae.2025.1564074

**Published:** 2025-02-07

**Authors:** Ellen V. Rothenberg, Ute Deichmann

**Affiliations:** ^1^ Division of Biology and Biological Engineering, California Institute of Technology, Pasadena, CA, United States; ^2^ Jacques Loeb Centre for the History and Philosophy of Science, Ben-Gurion University of the Negev, Beer Sheva, Israel

**Keywords:** chromatin modification and accessibility, DNA methylation, pioneers of epigenomics and modern chromatin research, chromatin interaction with transcription factors, epigenetic priming of development, hematopoietic epigenetics, transgenerational epigenetic inheritance

The term “epigenetic” has had its meaning change substantially since it was proposed over 80 years ago (see, e.g., Morange, 2002; Felsenfeld, 2014; Deichmann, 2016; Greally, 2018). Originally conceived broadly as the set of processes that generate phenotype from genotype over time in development (Waddington, 1942), it is currently used to mean the mechanisms demarcating different parts of the genome for differential gene activity in a given cellular context. We now have a highly sophisticated mechanistic understanding of the ways different genomic regions are marked by histone modification, DNA methylation, and chromatin remodelling in concert with differential local gene expression or local gene silencing. Other aspects of epigenetics include formation of dynamic protein condensates, chromatin looping, and shifts in intranuclear gene localization.

All these epigenetic mechanisms themselves depend, directly or indirectly, on the activities of genes that encode chromatin modifying enzymes, and they gain their target-gene specificity by the trans-acting, sequence-specific transcription factors and regulatory RNAs, which guide the chromatin modifiers to specific genomic locations. Of particular interest is the reciprocal way that transcription factors and chromatin epigenetic states affect each other’s action; sequence-specific transcription factor action is crucial to change gene expression for developmental progression, while chromatin states channel transcription factor activity and help to make development irreversible.

This Research Topic reveals some historical foundations of current chromatin research, and highlights the connection between events that are now called epigenetic, the genomic loci that encode epigenetic regulators, and the regulatory factors that guide them in development in different systems. It includes sophisticated case studies drawn from hematopoiesis, and an evaluation of the claims for epigenetic mechanisms in transgenerational inheritance in mammals.

Deichmann summarizes in-depth historical interviews with two pioneers of the field, Gary Felsenfeld and Sir Adrian Bird. Felsenfeld, who passed away at the beginning of May 2024, was a leader in many aspects of chromatin structural analysis. He specifically demonstrated the power of differential chromatin accessibility to identify active regulatory elements in the genome and investigated control of chromatin looping, the critical process that enables distal enhancers to interact with the correct promoters. Bird has long been a leader in the field of DNA methylation on CpG dinucleotides and their regulatory impact. This was one of the earliest-appreciated epigenetic marks, which first illustrated many of the paradigmatic aspects of epigenetic marking mechanisms in general. His work has revealed that a neurological disease, Rett syndrome, is caused by a mutation altering levels of a methyl-CpG binding protein that is an epigenetic modifier. As he noted, though: this syndrome is caused by mutations that change the DNA sequence. The primary change is the DNA sequence, and the secondary consequences are the epigenetic changes. It is thus an excellent example of the fact that genetics and epigenetics are closely linked.

Some of the most sophisticated work on epigenetic causality has been carried out in hematopoietic systems. Even before the sequencing of mouse and human genomes, intense work was already under way to determine the epigenetic mechanisms that control highly-specific expression of the genes that encode immunoglobulins in B cells and T-cell receptors in T cells (Nelsen et al., 1993; Stanhope-Baker et al., 1996; Nikolajczyk et al., 1999; Bergman et al., 2003; Cobb et al., 2006). This head start established these immunoreceptor loci as, arguably, the parts of the mammalian genome where epigenetic control is the best understood, as reviewed by Ma et al. These loci undergo highly cell type-specific DNA-level rearrangement in development as well as transcriptional activation. However, the chromatin structures, enhancers, transcription factors that work on the enhancers, and impacts of enhancer mutations on transcription in the correct lineages at the correct developmental times have been defined functionally in all of these loci with exceptional clarity, setting precedents that have subsequently proven to be of much wider generality. These genes continue to provide important insights: they include strong evidence for transcription factor combinatorial action and multiple well-demonstrated cases of temporal mismatches between chromatin opening and enhancer activation leading to transcription. These imply that additional levels of regulation still need to be explained.

Hematopoiesis more broadly has also been subjected to intense study to relate epigenetic mechanisms to developmentally controlled, lineage-specific cell type identity (e.g., Zeller et al., 2023; Yoshida et al., 2019; Goode et al., 2016; Javierre et al., 2016; González et al., 2015). The research paper by Maytum et al. takes a genome-wide view of the epigenomic transformations that accompany the earliest emergence of hematopoiesis. The DNA sequences in regions of chromatin undergoing epigenetic change give evidence that sequence-specific factors of the AP-1 transcription factor family, which normally become active in cells responding to acute signaling, are involved in opening chromatin at specific sites marking different developmental pathways. A paradox is that AP-1 is not cell type-specific; how can it guide distinct patterns of epigenomic change for different cell types? The authors present evidence that AP-1 gains distinct target site specificities from interaction with distinct collaborating factors. This study provides a vivid picture of the ways that in certain contexts, factors like AP-1 that normally mediate short-term signaling can have long term epigenetic effects in a cell lineage.

The hallmark feature of epigenetic mechanisms, collectively, is their ability to stabilize and perpetuate gene expression states across cellular generations. In the 1990s, it was proposed that epigenetic mechanisms can operate not only across cellular generations but also across organismal generations. Transgenerational epigenetic inheritance has been discussed as an urgent sociological issue, connected with possible very long-term impacts of factors ranging from teratogen exposure *in utero* to psychological stress in early life. A critical assessment of many of these studies is in Deichmann (2020). So far, transgenerational inheritance has not been clearly demonstrated for humans, and there is also no conclusive evidence for the influence of the environment on the epigenome (Bird). Many potential examples of epigenetic inheritance in humans are due to parental or intergenerational effects, in which the uterus is exposed to deleterious influences that directly affect the developing embryo and its germline, and DNA sequence changes as underlying cause for heritability are rarely excluded (Horsthemke, 2018). The state of the evidence and possibilities for transgenerational inheritance in mammals, with some examples also presented from nematodes and plants, are discussed in detail by Bird.

This Research Topic is hoped to provide context and provocations for much further work in this field.
